# Wolfram Syndrome: A Case Report and Review of Clinical Manifestations, Genetics Pathophysiology, and Potential Therapies

**DOI:** 10.1155/2018/9412676

**Published:** 2018-04-18

**Authors:** N. B. Toppings, J. M. McMillan, P. Y. B. Au, O. Suchowersky, L. E. Donovan

**Affiliations:** ^1^Department of Biological Sciences, University of Calgary, Calgary, AB, Canada; ^2^Department of Medicine, University of Calgary, Calgary, AB, Canada; ^3^Department of Medical Genetics, University of Calgary, Calgary, AB, Canada; ^4^Departments of Medicine, Medical Genetics and Pediatrics, University of Alberta, Edmonton, AB, Canada; ^5^Department of Medicine, Division of Endocrinology and Metabolism and Department of Obstetrics and Gynaecology, University of Calgary, Calgary, AB, Canada

## Abstract

**Background:**

Classical Wolfram syndrome (WS) is a rare autosomal recessive disorder caused by mutations in* WFS1, *a gene implicated in endoplasmic reticulum (ER) and mitochondrial function. WS is characterized by insulin-requiring diabetes mellitus and optic atrophy. A constellation of other features contributes to the acronym DIDMOAD (Diabetes Insipidus, Diabetes Mellitus, Optic Atrophy, and Deafness). This review seeks to raise awareness of this rare form of diabetes so that individuals with WS are identified and provided with appropriate care.

**Case:**

We describe a woman without risk factors for gestational or type 2 diabetes who presented with gestational diabetes (GDM) at the age of 39 years during her first and only pregnancy. Although she had optic atrophy since the age of 10 years, WS was not considered as her diagnosis until she presented with GDM. Biallelic mutations in* WFS1* were identified, supporting a diagnosis of classical WS.

**Conclusions:**

The distinct natural history, complications, and differences in management reinforce the importance of distinguishing WS from other forms of diabetes. Recent advances in the genetics and pathophysiology of WS have led to promising new therapeutic considerations that may preserve *β*-cell function and slow progressive neurological decline. Insight into the pathophysiology of WS may also inform strategies for *β*-cell preservation for individuals with type 1 and 2 diabetes.

## 1. Background

Wolfram syndrome (WS) is a form of monogenic diabetes that typically presents with diabetes mellitus in childhood and optic atrophy by the age of 16. The prevalence of WS has been estimated between 1 in 770,000 in the United Kingdom [1] and 1 in 100,000 in North America [2]. This entity was first described in 1938 by Wolfram and Wagener [3]. Many individuals with WS eventually develop diabetes insipidus and deafness, hence the acronym, DIDMOAD (Diabetes Insipidus, Diabetes Mellitus, Optic Atrophy, and Deafness). Additional morbidities include hypogonadism, infertility, hypopituitarism [4], cerebellar ataxia, peripheral neuropathy, dementia, psychiatric illness, and urinary tract problems [5–7].

Previously hypothesized to be a mitochondrial disorder, it is now known that classical WS is the result of autosomal recessive mutations affecting the* WFS1 *gene, which is implicated in endoplasmic reticulum (ER) function. Autosomal dominant mutations in* WFS1* have been reported to cause WS-like diseases characterized by diabetes, low frequency sensorineural hearing loss, psychiatric illness, variable optic atrophy, and* WSF1*-related low frequency sensorineural hearing loss [8–10].

This paper reviews recent advances in the understanding of the genetics and pathophysiology of WS and summarizes promising therapeutic management options directed at preserving *β*-cell function and slowing progressive decline in neurologic function.

## 2. Case Presentation

A 39-year-old woman of mixed Northern European descent was diagnosed with gestational diabetes (GDM) at sixteen weeks of gestation during her first and only pregnancy. Age was her only risk factor for GDM. She was treated with insulin, up to a maximum of 25 units per day, to maintain euglycaemia during pregnancy. Her pre-insulin-treatment A1c was 6.1% (43 mmol/mol) (upper limit normal range A1c < 6.1%, 43 mmol/mol). A healthy female neonate weighing 3374 g was delivered at 39 weeks of gestation by cesarean section, due to transverse lie. Three months postpartum, the patient met criteria for diabetes mellitus based on a 75 g oral glucose tolerance test; her 2-hour glucose value was 13 mmol/L (234 mg/dl) and fasting glucose 6.0 mmol/L (108 mg/dl). Following pregnancy, her daily insulin requirements dropped, to 6–12 units per day (0.12 to 0.24 units/kg/day).

Currently, 11 years after the birth of her daughter, her quarterly A1c values remain less than 7.0% (53 mmol/mol). Her 2-hour postprandial glucose values usually remain less than 6.5 mmol/L (117 mg/dl) despite only administering premeal insulin when her meals contain more than 60 g of carbohydrates. She consistently administers intermediate insulin at bedtime. She has never experienced diabetic ketoacidosis or severe hypoglycaemia requiring the assistance of another person. In the last year, she has begun to experience the need to drink water when swallowing food.

Her past medical history was significant for optic atrophy, diagnosed at age 10 which progressed to severe visual impairment over the following 8 years. From the age of 25 years, the patient complained of dizziness, unsteady gait with minor falls, and high-frequency sensorineural hearing loss.

Family history revealed one healthy sister, a brother with trisomy 21 who died at the age of 2 years from a congenital heart defect, and late onset deafness in her mother and maternal grandmother (in their seventies). Her father died at the age of 69 years of prostate cancer. There was no family history of diabetes mellitus or optic atrophy and no consanguinity.

On examination, her BMI was 17.8 kg/m^2^. She reported a low BMI throughout her life. She had bilateral optic atrophy with pale discs and impaired colour vision. Visual acuity was 20/400 in both eyes, with bilateral central scotomas. She had mild dysdiadochokinesia without dysmetria. She was unable to perform tandem gait for more than a few steps. She notably lacked typical findings associated with diabetes mellitus type 2, such as obesity, hypertension, and acanthosis nigricans.

Brain MRI revealed marked cerebellar vermian and hemispheric atrophy, as well as brainstem atrophy, particularly involving the pons. There was minimal change on MRI of her brain over a 10-year interval.

Antiglutamic acid decarboxylase and anti-islet cell antibodies were negative. Nutritional and vitamin deficiencies were ruled out with biochemical testing.

Genetic testing for autosomal dominant optic atrophy (*OPA1*) and spinocerebellar ataxia 1–8 and 17 were negative. Clinical sequencing of the* WFS1* gene was performed through the Casey Eye Institute. Two mutations were identified. The first variant was c.2590G>T (p.E864^*∗*^), a truncating mutation previously reported in patients affected with WS. The second variant was c.977C>T (p.A326V) and has been previously described in psychiatric patients [10] but has not yet been described as a known causative mutation in WS. This variant was predicted as likely damaging by some in silico prediction models (Polyphen 2) but also predicted as tolerated by other models (SIFT, PROVEAN). Targeted sequencing was performed for these two variants on a first-degree relative, and this family member was found to have only one of the two mutations. This segregation analysis therefore confirmed that the mutations were in trans in the patient and therefore affecting both maternally and paternally inherited alleles. Mitochondrial DNA sequencing had been considered to assess for maternally inherited diabetes and deafness (MIDD) but was not pursued once testing for* WFS1 *returned positive. A comparison between WS and MIDD is provided in Table 1.

An experimental therapeutic trial of a dipeptidyl peptidase-4 inhibitor (DPP-4) was offered in the hope of extending neurologic and *β*-cell function; however the patient declined this.

## 3. Discussion

This patient has typical features of classical WS, including optic atrophy, diabetes mellitus, hearing loss, and cerebellar ataxia. However, she is remarkable for her late age of onset of diabetes and slow progression of pancreatic *β*-cell loss. She has survived for more than 12 years past the average life-expectancy, as the typical median age of death for WS is 39 years (range 25–49 years) [1]. One of her genetic variants c.2590G>T (p.E864^*∗*^) has been reported previously in patients affected with WS and is a truncating loss of function mutation. Her other variant c.977C>T (p.A326V), resulting in a missense mutation, was described in psychiatric patients, but not as a causative mutation in WS [20]. However, this variant affects a highly conserved residue and is rare, with an allele frequency of less than 2.47 × 10^−5^; we feel this provides support for pathogenicity [21] and may be the cause of the mild phenotype. Thus, the presence of biallelic variants in* WFS1 *involving a known pathogenic truncating mutation and a rare missense variant, particularly in the context of her clinical findings, support the diagnosis of WS in our patient. It is possible, but less likely, that the true second pathogenic variant in WFS1 was missed with current sequencing technology.

### 3.1. Clinical Manifestations of WS

Diabetes mellitus is not the presenting clinical feature of WS in greater than 20% of patients. In nearly 15% of patients, the combination of both diabetes mellitus and optic atrophy is not yet present by 18 years of age [11]. In fact, less than one-third of patients meet the full clinical syndrome of Diabetes Insipidus, Diabetes Mellitus, Optic Atrophy, and Deafness, indicating that current clinical ascertainment criteria (early onset diabetes mellitus before 30 years of age and optic atrophy) do not ascertain all patients with WS [11] (Table 1).

Patients with WS may initially be misdiagnosed as type 1 diabetes mellitus. However, patients with WS are less likely to experience diabetic ketoacidosis and are half as likely to have microvascular complications as people with type 1 diabetes [13]. In contrast, the more common causes of morbidity and mortality in people with WS are neurological complications, such as central respiratory failure, ataxia, and neurogenic bladder [2, 13].

Glycaemic control tends to be better in those with WS than individuals with type 1 diabetes. In an age and diabetes duration-matched comparison between WS and type 1 diabetes mellitus patients, A1c was lower for WS, 7.72 ± 0.21% versus 8.99 ± 0.25%, respectively, *p* = 0.002 (60.9 mmol/mol versus 74.8 mmol/mol), as were total daily insulin requirements (0.71 ± 0.07 versus 0.88 ± 0.04 International Unit/kg/day, *p* = 0.0325) [13]. The lower daily insulin requirements per kg of body weight in WS patients suggest either greater pancreatic *β*-cell reserve or better insulin sensitivity when compared to matched individuals with type 1 diabetes. Our patient has very low insulin requirement suggesting good insulin sensitivity and considerable residual *β*-cell reserve.

Between 11 and 29 percent of WS patients encounter metabolic complications, such as severe hypoglycaemia causing coma or seizure. Severe hypoglycaemia was more commonly reported in WS patients with coexistent neurological symptoms (9 of 31) compared to WS patients without coexistent neurological symptoms (3 of 28) [5]. Thus, coexistent neurological dysfunction in patients with WS appears to predispose to the high rate of severe hypoglycaemia observed.

In addition to diabetes, other endocrinological abnormalities such as diabetes insipidus are present in approximately 38% [11]. Other abnormalities include primary gonadal atrophy in males and menstrual irregularities and delayed menarche in females [1]. Short stature and growth hormone deficiency have been reported, as has hypopituitarism, which are believed to be due to hypothalamic dysfunction [4].

In the largest cohort series to date, neurological symptoms were present in 53% of patients by an average age of 15 years. The majority of symptoms were related to the brainstem and cerebellum, specifically, cerebellar ataxia (45%), peripheral neuropathy (39%), cognitive impairment (32%), epilepsy (26%), and lastly dysarthria, dysphagia, and nystagmus in 10% [5]. Brain MRI was abnormal in 54%, including atrophy of the cerebrum, cerebellum, and brainstem [5] as was observed in the woman described here.

### 3.2. Genotype Phenotype Correlations in WS

Genotype phenotype correlations in WS are unclear, with many reported potentially pathogenic variants [22] and considerable phenotypic variability in patients. Therefore, predicting and prognosticating the natural history of disease for an individual patient is not recommended. However, trends are emerging. In 2013 De Heredia et al. systematically reviewed 412 published WS cases in the literature, of which 337 had confirmed* WFS1 *mutations [11]. Using a genotype classification scheme based on the predicted amount of residual expression of defective WFS1 protein, they suggested that patients with genotypes that are likely to lead to absent protein production were more likely to have earlier onset diabetes, and possibly earlier onset optic atrophy, than patients with residual protein expression [11]. Genotype phenotype correlations are less evident for other features. Our patient had later onset of diabetes, which may in part be due to the presence of a milder missense mutation.

### 3.3. Pathophysiology of WS

**Figure 1:**
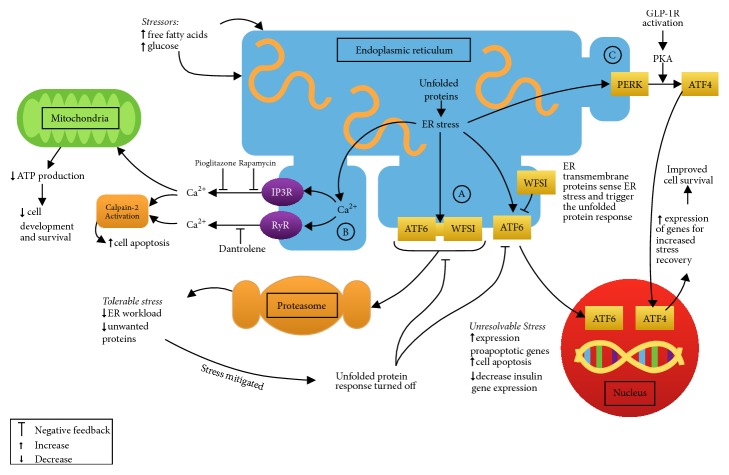
Hypothesized molecular pathophysiology of WS. Under situations of stress, such as hyperglycaemia and elevated free fatty acids levels, unfolded and misfolded proteins accumulate [31]. Endoplasmic reticulum (ER) transmembrane proteins sense the stress and activate the unfolded protein response (UPR) [31]. The UPR may culminate in either an adaptive response which decreases the workload on the ER or a maladaptive response (as occurs in chronic hyperglycaemia or WS) which culminates in cellular apoptosis [31]. *Ⓐ* In healthy cells, the transmembrane protein (WFS1) complexes with activating transcription factor-6 (ATF-6) and directs ATF-6 to ubiquitin-mediated proteasome degradation [31]. This serves to negatively regulate the UPR [31]. In WFS1 deficient cells ATF-6 is no longer under negative inhibition and is permitted to constitutively activate genes that promote cellular apoptosis and decrease insulin gene expression [31]. *Ⓑ* ER calcium channels, such as the ryanodine receptor (RyR), but most importantly the inositol triphosphate receptor (IP3R), permit efflux of calcium from the ER to the cytosol [16, 28]. It is believed that increased cytoplasmic calcium levels activate the calcium-dependent protease, calpain-2, which promotes cellular apoptosis [16, 34]. Potential therapeutic targets include molecules which inhibit calcium efflux from the ER, such as dantrolene, via inhibition of the RyR [35], and rapamycin and pioglitazone, via inhibition of IP3R [34]. In neuronal WS models, cytosolic calcium appears to be increased under resting conditions and reduced under stimulated conditions [28]. This disruption in cytoplasmic calcium homeostasis also dysregulates mitochondrial dynamics which leads to lower ATP levels [28]. This is thought to hinder neuronal development and survival [28]. *Ⓒ* Under periods of ER stress, pancreatic ER kinase (PERK), a transmembrane ER protein, becomes activated and through the action of protein kinase A (PKA) and cyclic-AMP (cAMP) results in the phosphorylation of translation initiation factor 2*α* (eIF2*α*) (not shown) [36]. This in turn results in increased production of activating transcription factor 4 (ATF4) which increases the expression of genes for ER stress recovery [31, 36, 37]. Furthermore, phosphorylated eIF2*α* leads to decreased overall protein synthesis and therefore to reduction in the ER protein load (not shown) [36]. Glucagon-like peptide-1 receptor (GLP-1R) activation, acting downstream of PERK, decreases the phosphorylation of eIF2*α* via the PKA/cAMP pathway, in order to ameliorate the decrease in protein synthesis that would otherwise occur (not shown) [36, 37]. This mechanism of action of GLP-1R activity facilitates a faster resumption of protein synthesis following ER stress (not shown) [36, 37].

*WFS1 *maps to chromosome 4p16.1 [22, 23]. The gene product (a transmembrane glycoprotein localized primarily to the ER [24]) functions to maintain homeostasis in the ER, the cellular organelle responsible for the folding of secretory proteins, such as insulin. Endocrine cells are particularly vulnerable to ER stress due to their rapid changes in secretory protein expression levels [25]. When ER homeostasis is disrupted, misfolded and unfolded proteins accumulate, leading to a state of ER stress [26]. The unfolded protein response is a response to ER stress in which cellular apoptosis may be triggered if the stress cannot be relieved [26] (Figure 1). ER stress is believed to play a role in *β*-cell dysfunction and apoptosis in type 1 and 2 diabetes mellitus and in other monogenetic forms of diabetes [27]. In WS, functional* WFS1* protein deficiency alters IP3R-mediated ER calcium release, disrupting cytoplasmic calcium homeostasis [28] (Figure 1). Additionally, calpain-2, a calcium-dependent proapoptotic cellular protease, may play a role in ER stress-induced apoptosis through increased cytoplasmic calcium levels [29–31] (Figure 1). Calcium-calpain-2 pathway overactivation is thought to contribute to pancreatic *β*-cell dysfunction and apoptosis in diseases such as type 2 diabetes mellitus and WS [30, 32]. Cagalinec et al. 2016 demonstrated that disruption in cytoplasmic calcium homeostasis in neurons also dysregulates mitochondrial dynamics which results in lower ATP levels. This is thought to hinder neuronal development and survival [28]. This likely explains the mitochondrial phenotype associated with WS. Interestingly, elevated free fatty acid levels, which are often present in type 2 diabetes mellitus and the metabolic syndrome, have been shown to promote activation of the calcium-calpain-2 pathway which promotes cellular apoptosis [33].

A second (ever rarer) type of WS (WS type 2) has a similar phenotype to classical WS. The causative gene for WS (type 2),* CISD2*, encodes an ER small protein, implicated in structural integrity and functional cross-talk between the ER and mitochondria [38]. Manifestations of WS (type 2) include diabetes mellitus, peptic ulcers, prolonged bleeding time, and neurodegenerative features [38–41]. The phenotypic overlap with classical WS is likely due to the overlapping function of the* CISD2* and* WFS1* gene products [7].

### 3.4. Promising Therapeutic Considerations for Those with WS

WS mutations lead to increased ER stress, altered cytoplasmic calcium, and dysregulation of mitochondria, which inhibits cellular growth and survival. Treatments which attempt to reduce ER stress may improve cell survival, notably, neural and pancreatic *β*-cell survival. In 2014, Lu et al. demonstrated that dantrolene could prevent apoptosis of WS patients' neural progenitor cells [34]. Dantrolene inhibits ryanodine receptors in the ER and functions to suppress efflux of calcium from the ER to the cytosol (Figure 1) [42]. Hepatotoxicity, even with sporadic short-term use, is a known side effect of this drug that may range from asymptomatic transaminase elevations to fulminant hepatic failure [16]. Previous reports of dantrolene-related fatal hepatotoxicity have been associated with daily doses greater than 300 mg/day [42, 43]. More recently it has been suggested that lower daily doses (i.e., <200 mg/day) may be safely used in patients without coexisting liver dysfunction or coingestion of hepatotoxic medications [16]. It is important to systematically evaluate dantrolene safety prior to it becoming usual care for WS because reduced cytosolic calcium in the setting of neuronal stimulation in WS* in vitro *neuronal models has been linked with poor mitochondrial function [28]. A phase 1 clinical trial is currently investigating safety of long term use (A Clinical Trial of Dantrolene Sodium in Pediatric and Adult Patients with Wolfram Syndrome, ClinicalTrials.gov, NCT02829268).

In 2006, Yusta et al. showed that a glucagon-like peptide-1 receptor (GLP-1R) agonist interfered with the ER unfolded protein response, resulting in decreased apoptotic signalling and increased cell survival (Figure 1) [44, 45]. In a mouse model of classical WS, the GLP-1R agonist exenatide effectively treated hyperglycaemia [35]. Treatment of a patient with WS (type 2) with exenatide was associated with a 70% reduction in daily insulin dose, improved glycaemic control, and a 7-fold increase in maximal insulin secretion [46]. Therefore GLP-1R agonists have potential as therapeutic agents in patients with WS because of their role in decreasing ER stress mediated pancreatic *β*-cell apoptosis. Another strategy would be to use a DPP-4 inhibitor of the enzyme that deactivates glucagon-like peptide-1 (GLP-1), thus increasing GLP-1 levels.

In 2009, the role of pioglitazone, a thiazolidinedione, was studied in WS knockout mice that ordinarily develop insulin-dependent diabetes by an average age of 8 weeks [47]. These mice exposed to pioglitazone were found to be protected from pancreatic *β*-cell apoptosis and to be almost completely protected from the development of diabetes mellitus [47]. It has been hypothesized that pioglitazone inhibits inositol triphosphate receptor (IP3R) release of calcium from the endoplasmic reticulum (Figure 1) [32]. Pioglitazone and other thiazolidinediones may have a therapeutic role in the management of patients with WS. However, recent concerns linking thiazolidinediones use with increased risk of heart failure [48], osteoporosis [36, 37], and bladder cancer [49] make them a less attractive option than GLP-1R agonists or DPP-4 inhibitors. Furthermore, Cagalinec et al. suggest that reducing cytosolic calcium has negative effects on mitochondrial dynamics [28].

*In vitro* models of WS (type 2) have shown the potential of iron chelation therapy to preserve neuronal cell and *β*-cell function [46]. Rapamycin is thought to reduce cytoplasmic calcium by a mechanism similar to pioglitazone (Figure 1) [32] but side effects and expense of rapamycin make it a less promising therapeutic option to investigate. Valproate has been shown to reduce ER stress-induced apoptosis in a model of diabetic nephropathy [50]. The molecular mechanisms for its diverse epigenetic effects on certain ER stress-related diseases have yet to be elucidated [51]. Valproate should be avoided in reproductive age women because of its known teratogenicity. We are unaware of any clinical trials of valproate in WS but it has been designated an orphan drug for the treatment of WS [52].

In addition to the targeted drug approaches described above, gene based therapies which include adeno-associated virus [53] and Clustered Regularly Interspaced Short Palindromic Repeats (CRISPR) technology [7] are also being pursued. Mesencephalic astrocyte-derived neurotrophic factor (MANF) is also being tested as a method of preserving and proliferating existing *β*-cells and neurons [7].

## 4. Conclusions

The woman described in this report has biallelic mutations in* WFS1*, supporting a diagnosis of classical WS. She developed diabetes at a much older age than is typical of classical WS, had a successful pregnancy, continues to have significant residual *β*-cell function, and has outlived the median age of death for WS by over 12 years. Her more favourable clinical course may in part be due to the presence of a milder missense mutation. Recent advances in the pathophysiology of WS have informed potential targeted therapeutics aimed at reducing associated morbidities. Treatments which attempt to reduce ER stress or improve mitochondrial function may improve neurologic and *β*-cell survival. As a greater understanding of this rare monogenic disorder is gained, valuable insight may be gained into other ER stress and mitochondrial disorders and *β*-cell preservation for individuals with type 1 and 2 diabetes and various neurodegenerative diseases.

## Figures and Tables

**Table 1:** Comparison of our patient to WS and MIDD.

	Case patient	Wolfram syndrome	Maternally inherited diabetes mellitus and deafness
Onset of diabetes mellitus	Diagnosed at the age of 39 years with gestational diabetes mellitus	Present in 98% [11]. Average age of diagnosis 6 years [1, 5]	Average age of diagnosis 37 years [12]

Anti-GAD and anti-islet cell antibodies	Absent	Absent	Absent

Diabetic ketoacidosis at presentation	Absent	3% [2]	No data reported

Retinal disease	Absent	35% diabetic retinopathy after 15 years [13]	Macular pattern dystrophy [14]

Renal disorders	Absent	8% [13]	Focal segmental glomerulosclerosis with hyalinised glomeruli, myocyte necrosis in afferent arterioles, and small arteries [15]

Optic atrophy	Diagnosed at the age of 10 years	Present in 82% [11]. Average age of diagnosis 10-11 years [5, 11]	No macular retinal dystrophy more common [14]

Sensorineural hearing loss	Diagnosed at the age of 25 years	Present in 48% [11]. Average age of diagnosis 16 years [1, 5]	Present in 75% Diagnosed between 2 and 61 years, frequently precedes diagnosis of DM, mean age of onset of hearing loss 33.2 years [15]

Diabetes insipidus	Absent	Present in classical WS 38% [11]. Average age of diagnosis 14-15 years [1, 5]. Absent in WS (type 2) [16]	Not routinely screened for

Neurological manifestations	Present, symptomatic by the age of 25 years	Present in 53% [5]. Average age of diagnosis 15 years [5] cerebellar ataxia, peripheral neuropathy, and dementia	Mitochondrial encephalomyopathy, lactic acidosis and stroke-like episodes (MELAS) [17]

Urological manifestations	Absent	Present in 19% [11]. Average age of diagnosis 12–20 years [1, 5]	Not routinely screened for

Gastrointestinal manifestations	Absent	Gastrointestinal dysmotility in 24% [1]. Severe gastrointestinal ulcer and bleeding in WS (type 2) [18]	Gastrointestinal dysmotility

Psychiatric manifestations	Absent	39% [5]	Depression, dementia, and psychosis [19]

Median age of death	n/a	39 years [1]	No reported data

Cause of death	n/a	Neurological complications [1, 2]	Lactic acidosis, renal failure [19]

## References

[1] Barrett T. G., Bundey S. E., Macleod A. F. (1995). Neurodegeneration and diabetes: UK nationwide study of Wolfram (DIDMOAD) syndrome. *The Lancet*.

[2] Kinsley B. T., Swift M., Dumont R. H., Swift R. G. (1995). Morbidity and mortality in the Wolfram syndrome. *Diabetes Care*.

[3] Wolfram D. J., Wagener H. P. (1938). Diabetes mellitus and simple optic atrophy among siblings: report of four cases. *Mayo Clinic Proceedings*.

[4] Medlej R., Wasson J., Baz P. (2004). Diabetes mellitus and optic atrophy: A study of Wolfram syndrome in the Lebanese population. *The Journal of Clinical Endocrinology & Metabolism*.

[5] Chaussenot A., Bannwarth S., Rouzier C. (2011). Neurologic features and genotype-phenotype correlation in Wolfram syndrome. *Annals of Neurology*.

[6] Swift R. G., Sadler D. B., Swift M. (1990). Psychiatric findings in Wolfram syndrome homozygotes. *The Lancet*.

[7] Urano F. (2016). Wolfram Syndrome: Diagnosis, Management, and Treatment. *Current Diabetes Reports*.

[8] Tranebjaerg L., Barrett T., Rendtorff N. D., Adam M. P., Ardinger H. H., Pagon R. A. (1993). WFS1-Related Disorders. *GeneReviews((R))*.

[9] Lesperance M. M., Hall J. W., San Agustin T. B., Leal S. M. (2003). Mutations in the Wolfram syndrome type 1 gene (WFS1) define a clinical entity of dominant low-frequency sensorineural hearing loss. *Archives of Otolaryngology—Head and Neck Surgery*.

[10] Bespalova I. N., Van Camp G., Bom S. J. H. (2001). Mutations in the Wolfram syndrome 1 gene (WFS1) are a common cause of low frequency sensorineural hearing loss. *Human Molecular Genetics*.

[11] De Heredia M. L., Clèries R., Nunes V. (2013). Genotypic classification of patients with Wolfram syndrome: Insights into the natural history of the disease and correlation with phenotype. *Genetics in Medicine*.

[12] Owen M. R., Doran E., Halestrap A. P. (2000). Evidence that metformin exerts its anti-diabetic effects through inhibition of complex 1 of the mitochondrial respiratory chain. *Biochemical Journal*.

[13] Cano A., Molines L., Valéro R. (2007). Microvascular diabetes complications in Wolfram syndrome (diabetes insipidus, diabetes mellitus, optic atrophy, and deafness [DIDMOAD]): An age- and duration-matched comparison with common type 1 diabetes. *Diabetes Care*.

[14] Massin P., Virally-Monod M., Violettes B. (1999). Prevalence of macular pattern dystrophy in maternally inherited diabetes and deafness. *Ophthalmology*.

[15] Guillausseau P.-J., Massin P., Dubois-LaForgue D. (2001). Maternally inherited diabetes and deafness: A multicenter study. *Annals of Internal Medicine*.

[16] Kim J. Y., Chun S., Bang M. S., Shin H.-I., Lee S.-U. (2011). Safety of low-dose oral dantrolene sodium on hepatic function. *Archives of Physical Medicine and Rehabilitation*.

[17] Suzuki S. (2004). Diabetes mellitus with mitochondrial gene mutations in Japan. *Annals of the New York Academy of Sciences*.

[18] Amr S., Heisey C., Zhang M. (2007). A homozygous mutation in a novel zinc-finger protein, ERIS, is responsible for Wolfram syndrome 2. *American Journal of Human Genetics*.

[19] Donovan L. E., Severin N. E. (2006). Maternally inherited diabetes and deafness in a North American kindred: Tips for making the diagnosis and review of unique management issues. *The Journal of Clinical Endocrinology & Metabolism*.

[20] Crawford J., Zielinski M. A., Fisher L. J., Sutherland G. R., Goldney R. D. (2002). Is there a relationship between Wolfram syndrome carrier status and suicide?. *American Journal of Medical Genetics Part B: Neuropsychiatric Genetics*.

[21] Gene: WFS1 http://exac.broadinstitute.org/gene/ENSG00000109501

[22] Inoue H., Tanizawa Y., Wasson J. (1998). A gene encoding a transmembrane protein is mutated in patients with diabetes mellitus and optic atrophy (Wolfram syndrome). *Nature Genetics*.

[23] Strom T. M., Hörtnagel K., Hofmann S. (1998). Diabetes insipidus, diabetes mellitus, optic atrophy and deafness (DIDMOAD) caused by mutations in a novel gene (wolframin) coding for a predicted transmembrane protein. *Human Molecular Genetics*.

[24] Takeda K., Inoue H., Tanizawa Y. (2001). WFS1 (Wolfram syndrome 1) gene product: Predominant subcellular localization to endoplasmic reticulum in cultured cells and neuronal expression in rat brain. *Human Molecular Genetics*.

[25] Ariyasu D., Yoshida H., Hasegawa Y. (2017). Endoplasmic reticulum (Er) stress and endocrine disorders. *International Journal of Molecular Sciences*.

[26] Ron D., Walter P. (2007). Signal integration in the endoplasmic reticulum unfolded protein response. *Nature Reviews Molecular Cell Biology*.

[27] Stoy J., Edghill E. L., Flanagan S. E. (2007). Insulin gene mutations as a cause of permanent neonatal diabetes. *Proceedings of the National Acadamy of Sciences of the United States of America*.

[28] Cagalinec M., Liiv M., Hodurova Z. (2016). Role of mitochondrial dynamics in neuronal development: mechanism for wolfram syndrome. *PLoS Biology*.

[29] Tan Y., Dourdin N., Wu C., de Veyra T., Elce J. S., Greer P. A. (2006). Ubiquitous calpains promote caspase-12 and JNK activation during endoplasmic reticulum stress-induced apoptosis. *The Journal of Biological Chemistry*.

[30] Huang C. J., Gurlo T., Haataja L. (2010). Calcium-activated calpain-2 is a mediator of beta cell dysfunction and apoptosis in type 2 diabetes. *The Journal of Biological Chemistry*.

[31] Nakagawa T., Yuan J. (2000). Cross-talk between two cysteine protease families: activation of caspase-12 by calpain in apoptosis. *The Journal of Cell Biology*.

[32] Hara T., Mahadevan J., Kanekura K., Hara M., Lu S., Urano F. (2014). Calcium efflux from the endoplasmic reticulum leads to *β*-cell death. *Endocrinology*.

[33] Cui W., Ma J., Wang X. (2013). Free fatty acid induces endoplasmic reticulum stress and apoptosis of *β*-cells by Ca^2+^/calpain-2 rathways. *PLoS ONE*.

[34] Lu S., Kanekura K., Hara T. (2014). A calcium-dependent protease as a potential therapeutic target for Wolfram syndrome. *Proceedings of the National Acadamy of Sciences of the United States of America*.

[35] Sedman T., Rünkorg K., Krass M. (2016). Exenatide Is an Effective Antihyperglycaemic Agent in a Mouse Model of Wolfram Syndrome 1. *Journal of Diabetes Research*.

[36] Meymeh R. H., Wooltorton E. (2007). Health and drug alerts: Diabetes drug pioglitazone (Actos): Risk of fracture. *Canadian Medical Association Journal*.

[37] Kahn S. E., Zinman B., Lachin J. M. (2008). Rosiglitazone-associated fractures in type 2 diabetes: an Analysis from A Diabetes Outcome Progression Trial (ADOPT). *Diabetes Care*.

[38] Rouzier C. C., Moore D., Delorme C. C. (2017). A novel CISD2 mutation associated with a classical Wolfram syndrome phenotype alters Ca2+ homeostasis and ER-mitochondria interactions. *Human Molecular Genetics*.

[39] El-Shanti H., Lidral A. C., Jarrah N., Druhan L., Ajlouni K. (2000). Homozygosity mapping identifies additional locus for Wolfram syndrome on chromosome 4q. *American Journal of Human Genetics*.

[40] Al-Sheyyab M., Jarrah N., Younis E. (2001). Bleeding tendency in Wolfram syndrome: A newly identified feature with phenotype genotype correlation. *European Journal of Pediatrics*.

[41] Mozzillo E., Delvecchio M., Carella M. (2014). A novel CISD2 intragenic deletion, optic neuropathy and platelet aggregation defect in Wolfram syndrome type 2. *BMC Medical Genetics*.

[42] Chan C. H. (1990). Dantrolene sodium and hepatic injury. *Neurology*.

[43] Utili R., Boitnott J. K., Zimmerman H. J. (1977). Dantrolene associated hepatic injury: incidence and character. *Gastroenterology*.

[44] Yusta B., Baggio L. L., Estall J. L. (2006). GLP-1 receptor activation improves *β* cell function and survival following induction of endoplasmic reticulum stress. *Cell Metabolism*.

[45] Wek R. C., Anthony T. G. (2006). EXtENDINg *β* cell survival by UPRegulating ATF4 translation. *Cell Metabolism*.

[46] Danielpur L., Sohn Y.-S., Karmi O. (2016). GLP-1-RA corrects mitochondrial labile iron accumulation and improves *β*-cell function in type 2 wolfram syndrome. *The Journal of Clinical Endocrinology & Metabolism*.

[47] Akiyama M., Hatanaka M., Ohta Y. (2009). Increased insulin demand promotes while pioglitazone prevents pancreatic beta cell apoptosis in Wfs1 knockout mice. *Diabetologia*.

[48] Nissen S. E., Wolski K. (2010). Rosiglitazone revisited: an updated meta-analysis of risk for myocardial infarction and cardiovascular mortality. *JAMA Internal Medicine*.

[49] Lewis J. D., Ferrara A., Peng T. (2011). Risk of bladder cancer among diabetic patients treated with pioglitazone: interim report of a longitudinal cohort study. *Diabetes Care*.

[50] Sun X.-Y., Qin H.-J., Zhang Z. (2016). Valproate attenuates diabetic nephropathy through inhibition of endoplasmic reticulum stress-induced apoptosis. *Molecular Medicine Reports*.

[51] Rakitin A. (2017). Does valproic acid have potential in the treatment of diabetes mellitus?. *Frontiers in Endocrinology*.

[52] (2015). *Public summary of opinion on orphan designation. Sodium valproate for the treatment of Wolfram Syndrome*.

[53] Hamel C., Jagodzinska J., Bonner-Wersinger D., Koks S., Seveno M., Delettre C. (2017). Advances in gene therapy for Wolfram syndrome. *Acta Ophthalmologica*.

